# Inhibition of USP14 and UCH37 deubiquitinating activity by b-AP15 as a potential therapy for tumors with *p53* deficiency

**DOI:** 10.1038/s41392-020-0143-9

**Published:** 2020-04-01

**Authors:** Yu-Shui Ma, Xiao-Feng Wang, Fei Yu, Ting-Miao Wu, Ji-Bin Liu, Yun-Jie Zhang, Qing Xia, Zong-Yuan Jiang, Qin-Lu Lin, Da Fu

**Affiliations:** 1 Central Laboratory for Medical Research, Shanghai Tenth People’s Hospital, Tongji University School of Medicine, 200072 Shanghai, China; 2 Cancer Institute, Nantong Tumor Hospital, 226631 Nantong, China; 3 Department of Nuclear Medicine, Shanghai Tenth People’s Hospital, Tongji University School of Medicine, 200072 Shanghai, China; 4 Department of Radiology, The Fourth Affiliated Hospital of Anhui Medical University, 230012 Hefei, China; 5 Department of Orthopedics, Zhongshan Hospital, Fudan University, 200032 Shanghai, China; 6 Department of Hand Surgery, Shenzhen Longhua District People’s Hospital, 518109 Shenzhen, China; 7 National Engineering Laboratory for Rice and By-product Deep Processing, College of Food Science and Engineering, Central South University of Forestry and Technology, 410004 Changsha, China

**Keywords:** Oncogenes, Drug development

**Dear Editor,**

Tumor-suppressor protein p53 is important for cell function and genome integrity.^1^ The decrease of p53 protein is a common feature of human malignant tumors, which leads to the deficiency of cell cycle detection point control and apoptosis induction. An enormous amount of research effort goes into small molecules that regulate p53, including wild-type repair of mutant p53 gene and interruption of the binding between p53 and an E3-ubiquitin ligase Mdm2 to prevent ubiquitination degradation of p53 and rescue the protein level of p53.^2^ However, it is not clear whether it is necessary to continue to inhibit the ubiquitin activity of proteasome in order to restore the p53 protein level.

To explore this issue, we utilized p53 knockout mice,^3^ in which the p53 gene of germ line is disrupted and induces the deficiency of p53 in all organs and tissues of adult mice, to screen specific small molecule inhibitors for p53-deficient tumors. Most of the adult *p53*^−/−^ mice died of malignant lymphomas of thymus^3^ and heterozygous *p53*^+/−^ mice develop sarcoma (including soft tissue sarcoma and osteosarcoma) at a late age (between 10 and 12 months) (Fig. 1a, b).

**Fig. 1:**
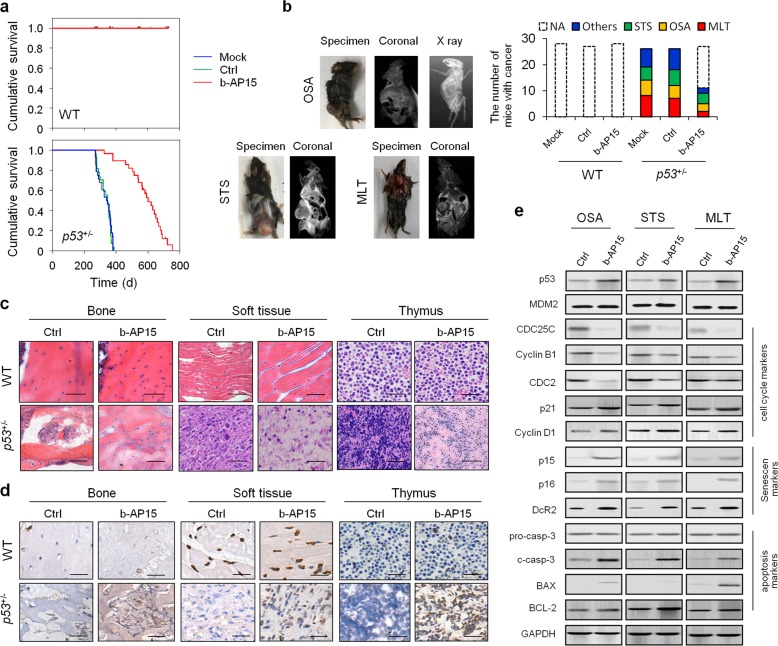
b-AP15 resulted in durable tumor regressions in p53-deficient mice. **a** Kaplan–Meier survival analysis was used to evaluate the treatment effect of b-AP15 in WT and heterozygous *p53*^+/−^ mice for OS. **b** X-ray, micro-CT, and MRI analysis and type of primary tumors in p53-deficient mice. The effect of b-AP15 in WT and heterozygous *p53*^+/−^ mice on the number of types of cancer was quantified. H&E staining analysis (**c**) and IHC staining for p53 (**d**) of normal or primary tumors in the bone, soft tissue, and thymus in WT or *p53*-deficient mice with or without treatment of b-AP15. **e** WB was used to measure the effect of b-AP15 on the protein level of cell cycle-, senescent-, and apoptosis-associated markers in heterozygous *p53*^+/−^ mice. Ctrl control, MLT malignant lymphomas of thymus, NA not applicable, OSA osteosarcoma, STS soft tissue sarcoma, WT wild type

We previously confirmed that a specific USP14 and UCH37 inhibitor b-AP15^4^ inhibited tumor cell growth and induced apoptosis and in vitro (data not shown). Hence, in this study, we further investigated the effect of b-AP15 on inhibiting tumor growth in heterozygous *p53*^+/−^ mice in vivo. A significant prolong of overall survival in *p53*^+/−^ mice was observed after b-AP15 administration (*P* < 0.0001) (Fig. 1a). Weight of mice in the control group was observed to significantly decrease. In contrast, the body weight and main organ weights (e.g., liver and lung) of mice in the b-AP15-treated group restored to normal weight. The number of mice with tumors was found to obviously decrease (Fig. 1b), and the tissue morphology was partially restored in heterozygous *p53*^+/−^ mice group after b-AP15 treatment (Fig. 1c).

Treatment with b-AP15 significantly increased the protein levels of p53 (Fig. 1d) and p21. However, the protein levels of G2/M phase cell cycle regulatory protein cell division cycle (CDC) 25C, its downstream protein cyclin B1 and CDC2 decreased obviously (Fig. 1e). Moreover, the expression of the p53 regulator MDM2 is not affected in tumors following mice treatment with b-AP15 (Fig. 1e). The protein levels of senescent markers, including the cyclin-dependent kinase inhibitors p16-Ink4a, p15-Ink4b, and decoy receptor 2, increased in senescent pre-neoplastic lesions in *p53*^+/−^ mice with b-AP15 treatment (Fig. 1e). The protein levels of cell apoptosis markers, including BCL-2, BAX, and cleaved caspase-3, are induced after p53 restoration in *p53*^+/−^ mice in vivo (Fig. 1e).

Deubiquitination enzyme UCH37 interacts with substrate proteins and then deubiquitinates substrates to inhibit their ubiquitination degradation in 19S regulatory particle by ubiquitin–proteasome pathway (UPP).^5^ Next, the candidate interacting proteins and potential substrates of UCH37 was explored through a yeast two-hybrid system and mass spectrometry after immunoprecipitation (Supplementary Tables S1 and S2). A few previously reported proteasome regulatory proteins, including HAUS augmin-like complex subunit 7 (HAUS7), non-ATPase 13 (RPN13), and RPN10,^6^ were identified. Besides, COPS5, a master regulator in the cells,^7^ was identified as an candidate interacting protein of UCH37 (Supplementary Tables S1 and S2).

The interaction of UCH37 and COPS5 in 293T cells, in which both proteins were overexpressed, was confirmed using immunoprecipitation analysis by either anti-HA (Fig. S1a) or anti-Flag antibody (Fig. S1b). Moreover, the endogenous UCH37 interaction with endogenous COPS5 in U2OS cells (Supplementary Fig. S1c, d) was also demonstrated. Cell immunofluorescence and co-focusing experiment further strengthened the evidence linking UCH37 with COPS5 (Supplementary Fig. S1e). Moreover, the UCH domain and UCH with a 21-residue active-site crossover loop (ASCL) region interacted with COPS5; however, neither ASCL nor the C-terminal domain alone interacted with COPS5 (Supplementary Fig. S1f, g).

We detected the COPS5 protein level in various types of tumors in *p53*^+/−^ mice and found a significant increase. However, the protein level of COPS5 decreased after b-AP15 treatment, and there is a remarkable negative correlation between COPS5 and p53 protein level (Supplementary Fig. S1l, m).

We further found that UCH37 significantly upregulated the protein level of COPS5, but there is no obvious change for other candidate interacting protein, including RPN10, HAUS7, and RPN13 (Supplementary Fig. S1h, i). Moreover, the COPS5 protein level significantly decreased after knockdown of UCH37 and USP14 or b-AP15 treatment (Supplementary Fig. S1j, k).

UCH37 overexpression decreased the ubiquitination level of COPS5 (Supplementary Fig. S2a); in contrast, ubiquitination COPS5 significantly increased after treatment with b-AP15 (Supplementary Fig. S2b) in vitro and the protein level of COPS5 decreased in vivo in heterozygous *p53*^+/−^ mice (Supplementary Fig. S2c).

COPS5 was reported to induce protein ubiquitination degradation by factors including cell cycle inhibitor p21 and tumor-suppressor p53^8^ to induce an increase in cell proliferation. We found that p53 protein level was rescued after knockdown of COPS5 and induced by UCH37 and USP14 overexpression (Supplementary Fig. S2d). Similarly, knockdown of UCH37 and USP14 (Supplementary Fig. S2e) or b-AP15 treatment (Supplementary Fig. S2f) rescued p53 protein, induced by COPS5 overexpression, and enhanced the activity of transfected luciferase reporter plasmids for p53, Bax, and p21 expression (Supplementary Fig. S2g) and protein levels (Supplementary Fig. S2h) of p53 downstream target genes BAX and p21.

The nuclear and cytoplasmic co-localization between COPS5 and p53 (Supplementary Fig. S2i, j) suggests a mechanism of COPS5 and p53 interaction and relocalization of p53 from the nucleus to the cytoplasm, and treatment of MG132, a potent proteasome inhibitor, significantly inhibited proteasome-dependent protein degradation of p53 and enhanced its levels in cytoplasm, which indicated the degradation of p53 induced by COPS5 (Supplementary Fig. S2k). Treatment of leptomycin B (Supplementary Fig. S2l, m) inhibited signal-mediated nuclear export by direct binding to chromosome region maintenance 1 (CRM1), RNA interference of CRM1 (Supplementary Fig. S2n), or expression of COPS5 without nuclear export signal (NES) (COPS5^ΔNES^) (Supplementary Fig. S2o), arrested p53 nuclear export, and induced nuclear accumulation. Rpn13, the proteasomal receptor for Uch37 in the proteasome 19S regulatory particle, can activate UCH37 by disrupting dimerization.^9^ USP14 binds to the regulatory particle Rpn1 to release its catalytic USP domain and polyubiquitin chains of substrate protein.^10^ Overexpression of Rpn13 or Rpn1 upregulated the COPS5 protein levels and downregulated the p53 protein levels (Supplementary Fig. S2p). In contrast, knockdown of Rpn13 or Rpn1 upregulated the p53 protein levels and induced its nuclear accumulation (Supplementary Fig. S2q, r). Moreover, b-AP15 treatment rescued the p53 protein level induced by Rpn13 or Rpn1 overexpression (Supplementary Fig. S2p).

In conclusion, our results showed that treatment of b-AP15 rescued the protein level of p53 and blocked its nuclear export and ubiquitination degradation induced in UPP by Rpn13- and Rpn1-mediated and UCH37-dependent COPS5 deubiquitylation.

## Supplementary information

Supplementary material
