# Supplementary material for: Inhibition of USP14 and UCH37 deubiquitinating activity by b-AP15 as a potential therapy for tumors with p53 deficiency
Source: Signal Transduct Target Ther. 2020 Apr 1;5:30. doi: 10.1038/s41392-020-0143-9 (PMC7109122; doi:10.1038/s41392-020-0143-9)
Supplement: Supplementary file 1 — Supplementary material [file 41392_2020_143_MOESM1_ESM.docx]

**SUPPLEMENTAL INFORMATION**

**Inhibition of USP14 and UCH37 deubiquitinating activity by b-AP15 as a potential therapy for tumors with *p53* deficiency**

Supplemental information contains online methods, one supplemental figure and legend, and two tables.

**ONLINE METHODS**

**Animal models and genotyping**

All experimental procedures were approved by the Institutional Animal Care and Use Committee (IACUC) guidelines at Tongji University School of Medicine (SYDW-19-215).

The *p53*^-/-^ mice in C57BL/6 background, purchased from Jackson Laboratory, were crossed with WT mice and the resulting mice were further intercrossed to generate *p53*^+/−^ mice [1]. Genomic DNA from tail biopsies was genotyped by PCR. The *p53*^+/−^ mice were used to observe the spontaneous tumor formation, treatment of b-AP15 and primary cell cultures. b-AP15 (5mg/kg, 1%DMSO + 30%PEG300 + 1%Tween80 + ddH_2_O) was given i.p. twice a week for the number of days indicated. b-AP15 was purchased from SelleckChem. Mice without any treatment were used as the mock, and mice treated with vehicle (1%DMSO + 30%PEG300 + 1%Tween80 + ddH_2_O) were used as the control.

All mice were monitored by X-way, MRI or micro-CT diagnosis for tumor phenotypes weekly up to the age of 24 months before all of the surviving animals were sacrificed. The body and main organs (liver and lung) weight measurements were performed to collect the data when the mouse was died or up to the age of 24 months before all of the surviving animals were sacrificed. Moribund animals or those mice developing obvious tumors before this end point were also sacrificed and necropsied. The tumors were placed in 10% neutral buffered formalin for further histopathological analysis. Tumor histological type was independently confirmed by two experienced pathologists and tumor volume was calculated using the following formula: volume = length × width^2^ × 0.52. All cell mitotic figures within each tumor were counted and are presented as number of mitotic figures per unit area (cm^2^).

**Cell lines**

Human osteosarcoma epithelial cell lines U2OS, mouse B lymphoma cell WEH1-231 and human HEK293T cell lines were purchased from the Cell Bank of the Chinese Academy of Sciences (Shanghai, China), and cultured in DMEM media (Invitrogen, Carlsbad, USA) and supplemented with 10 % (v/v) fetal bovine serum (FBS), 100 U/ml penicillin, and 100 mg/ml streptomycin. Cell lines were routinely tested for mycoplasma contamination, and have been authenticated with short-tandem repeat analysis. Cell culture was conducted at 37 °C in a humidified 5% CO_2_ incubator.

**Plasmid construction and transfection**

Overexpression of USP14, UCH37, UCH37-Flag, UCH37^1-142^-Flag, UCH37^1-155^-Flag, UCH37^142-155^-Flag, UCH37^142-328^-Flag, UCH37^155-328^-Flag, Rpn13, Rpn1, Flag-p53, COPS5, HA-COPS5, COPS5^ΔNES^ was performed using the pMSCV retroviral plasmid. All constructs were confirmed by PCR and Sanger sequencing. The plasmids were transiently transfected into target cells with Lipofectamin 2000 (Life Technologies, Gaithersburg, MD). Cells were transiently transfected with genome control short interfering RNA (siRNA) of CRM1 and harvested 48 hours posttransfection, followed by analysis using immunoblotting assay.

To generate stable cell lines with specific gene overexpression or knockdown, the plasmids were packaged into retroviruses with the amphotropic Phoenix packaging cell line and infected into target cells, followed by puromycin/hygromycin selection of infected cells. USP14, UCH37, Rpn13 or Rpn1-knockdown cell lines were generated using short hairpin RNAs and retroviral transduction. Short hairpin RNA (shRNA) a random sequence was set up as a control. For the transfection of siCRM1, U2OS were seeded at 1.2 × 10^5^/well onto 6-well plates overnight. Transfections were performed with 16.5 nM siCRM1 according to the manufacturer's instructions. The transfected cells were harvested at 48 h posttransfection for further assays.

**Western blot**

Total proteins were extracted from cells following the standard protocol [2]. Nuclear and cytoplasmic proteins were separated by Cytoplasmic & Nuclear Extraction Kit (invent; sc-003). Protein concentration was measured using the BCA protein assay kit (Thermo Scientific; 23225). The primary antibodies used in this study were as follows: GAPDH (cat. ab8245), UCH37 (cat. ab133508), USP14 (cat. ab137432), COPS5 (cat. ab210538), Rpn13 (cat. ab157218), Rpn1 (cat. ab198508), p27 (cat. ab32034), DcR2 (cat. ab2019), CDC25C (cat. ab32444), CDC2 (cat. ab18), cleaved caspase-3 (cat. ab2302), pro-caspase-3 (cat. ab13585), BAX (cat. ab32503), BCL-2 (cat. ab32124), Cyclin E1 (cat. ab33911), E2F7 (cat. ab56022), p15 (cat. ab53034), HAUS7 (cat. ab192616), PARP1 (cat. ab32064), HA (cat. ab18181), Cyclin B1 (cat. ab72) and p53 (cat. ab26) from Abcam, AP-1 (cat. 9165), p21 (cat. 2947), p16 (cat. 92803), Beclin-1 (cat. 3495), PSMD4 (cat. 3336), ADRM1 (cat. 12019), myc (cat. 2276) and Histone (cat. 3638) from Cell Signaling and Cyclin D1 (cat. 554181) from BD. The goat anti-rabbit IgG (Merck) and goat anti-mouse IgG (Merck) antibodies were used for western blot analyses. Antibody dilutions were 1:1,000 for primary antibodies and 1:5,000 for secondary antibodies in western blotting. Data are representation of 3-4 independent experiments.

**Histology and immunohistochemistry (IHC) analysis**

Standard IHC and H&E staining were used to evaluate protein expression levels in tumor samples. Tissues from mice were flushed and fixed in 4% formaldehyde in PBS for 24 h. Samples were then dehydrated and embedded in paraffin, sectioned at 5 μM and processed for H&E staining. The primary antibodies were: Ki-67 (Abcam, ab156956), Beclin-1 (Abcam, ab62557), p53 (Abcam, ab1101), UCH37 (Abcam, ab236002), COPS5 (Abcam, ab12323), USP14 (Cell Signaling, 11931), and cleaved-caspase3 (Cell Signaling, 9661). Staining was visualized with ABC Kit Vectastain Elite (Vector) or TSA kit (Invitrogen). Serial sections were stained in parallel with the primary antibody replaced by PBS as controls.

**Immunoprecipitation and immunoblotting**

For immunoprecipitation assays, cells were pretreated MG132, IU1 or b-AP15 for the previously indicated time periods, and lysed with HEPES lysis buffer (20 mM HEPES, pH 7.2, 50 mM NaCl, 0.5% NP-40, 1 mM NaF and 1 mM dithiothreitol) supplemented with protease-inhibitor cocktail (Roche). Immunoprecipitations were performed using the indicated primary antibody and protein A/G agarose beads (Roche) at 4 °C. Both lysates and immunoprecipitates were examined using anti-Ub, anti-Flag or anti-HA primary antibodies (Cell Signaling) and the related secondary antibody followed by detection with the chemiluminescence substrate (Millipore). For immunoblotting, total proteins were extracted from cells following the standard protocol. Cytomembrane free lysate were separated from cells by Native Membrane Protein Extraction Kit (Millipore; 444810). Nuclear and cytoplasmic proteins were separated by Cytoplasmic & Nuclear Extraction Kit (Invent; sc-003) [3]. Protein concentration was measured using the BCA protein assay kit (Thermo Scientific; 23225).

**Luciferase reporter assays**

Human p21 and Bax 3’-UTR oligonucleotides were subcloned into the XhoI and NotI sites of the pGL3 luciferase reporter plasmid (Promega, Madison, WI, USA). For the luciferase assay, U2OS cells were seeded into 24-well plates and cultured for 24 h, after which cells were co-transfected with reporter plasmid. Then 48 h after transfection, the luciferase assay was performed using the Dual-Luciferase Kit (Promega, Madison, WI).

**Yeast two hybrid screening**

Yeast two-hybrid screening was carried out using a GAL4-based yeast two-hybrid system (MATCHMAKER Two-Hybrid System 3; Clontech, Palo Alto, CA); screening and assays were performed following the manufacturer’s instruction (Clontech) [4].

Human UCH37 cDNA was amplified by PCR; the PCR fragment was then digested with NdeI and BamHI, and inserted into the pGBKT7 vector (Clontech) to generate a construct of mouse UCH37 cDNA fused inframe to the GAL4 DNA-binding domain (BD) (amino acids 1-147 of GAL4) as the bait. The vector was then transformed into yeast strain Y187 and the transformants were lated on dropout medium lacking tryptophan (SD/Trp) because the pGBKT7 vector had a selectable TRP1 marker.

The pre-transformed Human Brain Matchmaker cDNA Library in pGADT7 vector was purchased from Clontech. For yeast two-hybrid screening, the mating reaction between Y187 transformed with pGBKT7-UCH37 construct and AH109 pre-transformed with human brain library was performed and selected on Quadruple Drop Out (stringent selection) medium. Positive clones were further tested on medium containing X-alpha-Gal, which tests alphaglactosidase activated by positive UCH37-target protein interaction. The pGADT7 plasmids encoding the library clones were isolated and sequenced using an ABI 3100 Genetic Analyzer (Applied Biosystems, Foster city, CA), and homology searches against database sequences were performed using the BLAST algorithm on National Center for Biotechnology Information [5].

**Mass spectrometry**

Pellets of U2OS cell expressing Flag-UCH37 from two 150-mm plates were lysed in 50 mM HEPES-KOH (pH8.0), 100 mM KCl, 2 mM EDTA, 0.1% NP-40, 10% glycerol and affinity-purified using Flag-M2 magnetic beads (Sigma-Aldrich). Subsequently, digestion with trypsin (Worthington, Columbus) was performed on-beads. For liquid chromatography-tandem mass spectrometry analysis, peptides were reconstituted in 5% formic acid and loaded onto a 12–15-cm fused silica column with pulled tip packed with C18 reversed-phase material. Peptides were analysed using an LTQ-Orbitrap Velos (Thermo Scientific) or a 6600 Triple TOF (AB SCIEX, Framingham) coupled to an Eksigent NanoLC-Ultra HPLC system and a nano-electrospray ion source (Proxeon Biosystems, Thermo Fisher Scientific). Peptides were eluted from the column using a 90–100-min gradient of acetonitrile in 0.1% formic acid. The lyophilized peptide mixture was re-suspended in water with 0.1% formic acid (v/v) and its content was estimated by UV light spectral density at 280 nm, then 3 μg of the digest peptides were analyzed by nano-liquid chromatography-tandem mass spectrometry (LC-MS/MS) on LTQ Orbitrap Velos Pro mass spectrometer [6]. Raw data was processed by Maxquant software (1.3) and then used for database and spectral library searching using Andromeda peptide search engines. The Maxquant peptide and protein quantiﬁcation results files were imported into Perseus software (version 1.5.1.6) for further analysis [7, 8].

**RNA extraction and quantitative PCR (qPCR)**

RNA was extracted using TRIzol (ThermoFisher Scientific) following the manufacture’s protocol, and then subjected to cDNA synthesis using iScript kit (Bio-rad). RNA concentration was measured using a NanoDrop2000 spectrophotometer (Thermo Fisher Scientific, Waltham, MA, USA). Electrophoresis on 1.5% denaturing agarose gels was performed to evaluate the quality of all RNA specimens. The cDNA was obtained from total RNA by reverse transcription, and the final RNA concentration used in the quantitative PCR reaction was 10 ng, Real-time PCR was performed using the iTag universal SYBR Green kit (Bio-rad) and subsequently analyzed in a CFX Connect system (Bio-rad). Sequences of PCR primers are as following: GAPDH: Forward, 5'-ACCCAGAAGACTGTGGATGG-3', Reverse, 5'-TTCTAGACGGCAGGTCAGGT-3'; USP14: Forward, 5'-GGCGTGTGGAGATGTATAAC-3, Reverse, 5'-CAGCTCAGCACTATCCAGAC-3' and UCH37, Forward, 5'-GTCATTTGCTGTGGGTGATG-3', Reverse, 5'-AGAAGGAGGGAACGAATGGG-3'. GAPDH were used as the endogenous controls, and the 2^-ΔΔCT^ method was used to analyze expression levels [9].

**Imaging**

In the animal studies, we used the dual tube/detector micro-CT system that has been described in detail elsewhere [10]. The x-ray parameters were 80 kVp, 160 mA, and 10ms per exposure, and the radiation dose associated with the micro-CT scan was 16 cGy. MRI experiments were performed on a Bruker BioSpec 7.0 Tesla 30 cm clear bore USR (Ultra Shielded Refrigerated) horizontal bore Superconducting Magnet System [11]. The Bruker-made 23-mm ID birdcage volume radiofrequency coil was used for both radiofrequency excitation and receiving. Animals were anesthetized throughout the imaging procedure through the inhalation of a mixture of 1.5% isoflurane into medically supplied air.

**Statistical analysis**

Measurement data was expressed as mean ± S.D. (standard deviation). Categorical data were reported as numbers and percentages. Analysis of two samples was performed with unpaired two-tailed Student t test for equal variance, or t test with Welch’s correction for heterogeneity of variance. The chi-square test was used to evaluate the difference among different groups. Univariate survival analysis of overall survival was carried out using the Kaplan-Meier method. Spearman's correlation coefficient was used to test the relationship of two independent groups. All calculations were performed with the Prism 6.0 GraphPad or SPSS 20.0 software program (SPSS Inc, Chicago, IL, USA). The level of significance was chosen as *P* < 0.05.

**Supplementary References**

1. Jacks T, Remington L, Williams BO, Schmitt EM, Halachmi S, Bronson RT, *et al*. Tumor spectrum analysis in p53-mutant mice. Curr Biol. 1994, 4(1): 1-7.
2. Ma YS, Huang T, Zhong XM, Zhang HW, Cong XL, Xu H, *et al*. Proteogenomic characterization and comprehensive integrative genomic analysis of human colorectal cancer liver metastasis. Mol Cancer. 2018, 17(1): 139.
3. Ma YS, Lv ZW, Yu F, Chang ZY, Cong XL, Zhong XM, *et al*. MiRNA-302a/d inhibits the self-renewal capability and cell cycle entry of liver cancer stem cells by targeting the E2F7/AKT axis. J Exp Clin Cancer Res. 2018, 37(1): 252.
4. Choi YS, Hoon Jeong J, Min HK, Jung HJ, Hwang D, Lee SW, *et al*. Shot-gun proteomic analysis of mitochondrial D-loop DNA binding proteins: identification of mitochondrial histones. Mol Biosyst. 2011, 7(5): 1523-36.
5. Yu Z, Liu N, Wang Y, Li X, Wang X. Identification of neuroglobin-interacting proteins using yeast two-hybrid screening. Neuroscience. 2012, 200: 99-105.
6. Tian XP, Jin XH, Li M, Huang WJ, Xie D, Zhang JX. The depletion of PinX1 involved in the tumorigenesis of non-small cell lung cancer promotes cell proliferation via p15/cyclin D1 pathway. Mol Cancer. 2017, 16(1): 74.
7. Luo P, Lu G, Fan LL, Zhong X, Yang H, Xie R, *et al*. Dysregulation of TMPRSS3 and TNFRSF11B correlates with tumorigenesis and poor prognosis in patients with breast cancer. Oncol Rep. 2017, 37(4): 2057-2062.
8. Sun X, Zhang H, Luo L, Zhong K, Ma Y, Fan L, *et al*. Comparative proteomic profiling identifies potential prognostic factors for human clear cell renal cell carcinoma. Oncol Rep. 2016, 36(6): 3131-3138.
9. Livak KJ, Schmittgen TD. Analysis of relative gene expression data using real-time quantitative PCR and the 2^-ΔΔCt^ method. Methods, 2001. 25(4): 402‑408.
10. Badea CT, Johnston S, Johnson B, Lin M, Hedlund LW, Johnson GA. A dual micro-CT system for small animal imaging. Proc SPIE. 2018, 6913: 691342.
11. Ni J, Ramkissoon SH, Xie S, Goel S, Stover DG, Guo H, *et al*. Combination inhibition of PI3K and mTORC1 yields durable remissions in orthotopic patient-derived xenografts of HER2-positive breast cancer brain metastases. Nat Med. 2016, 22(7): 723–726.

**Supplementary figure and legend**

**
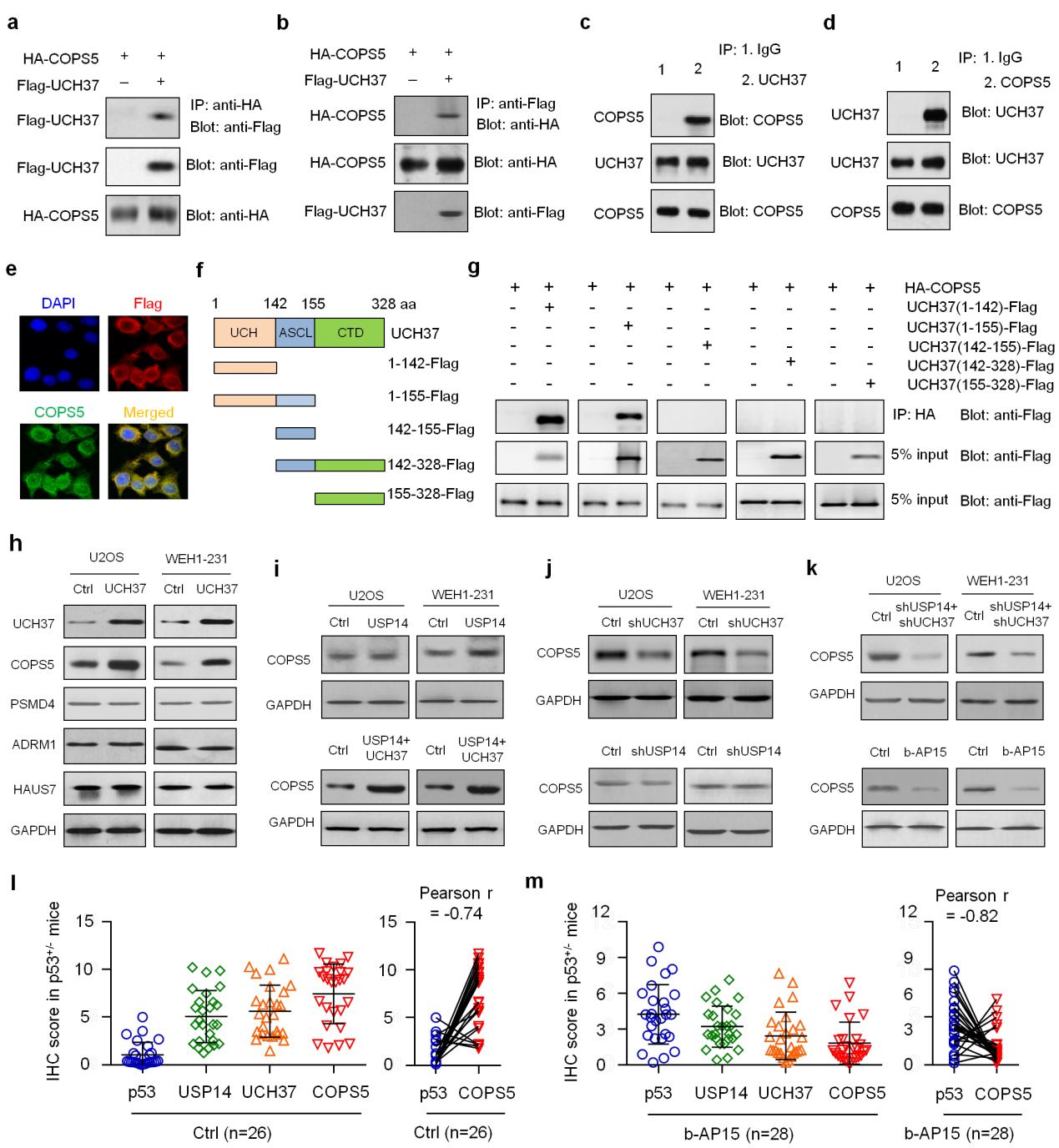
Supplementary Figure 1****. Identification of UCH37-interacting proteins.** (a, b) Immunoprecipitation analysis by either anti-HA (a) and anti-Flag antibody (b) after HA-COPS5 together with Flag-UCH37 expression plasmid was co-transfected in 293T cells. (c, d) Western blotting was used to detect UCH37 and COPS5 in U2O3 cells after co-IP of UCH37 (c) and COPS5 antibody (d). (e) Immunofluorescence technique was used to investigate the interaction of exogenous COPS5 (red) with UCH37 (green). U2O3 cells were cultured on cover slips and transfected with plasmid pFlag-UCH37. The nucleus was stained with DAPI (blue). (f) Schematic representation of UCH37 deletion mutant constructs. (g) U2OS cells were transfected with different UCH37 deletion mutants. Cell lysates were extracted, and proteins from lysates were subjected to Western blot using the indicated antibodies. ASCL, 21-residue active-site crossover loop; CDT, c-terminal domain;c-terminal domain; UCH, ubiquitin C-terminal hydrolase. (h) Western blotting was used to quantify candidates of UCH37-interacting protein level in U2O3 and WEH1-231 cells after UCH37 overexpression. (i) Western blotting was used to quantify COPS5 protein level in U2O3 and WEH1-231 cells after USP14 or USP14 and UCH37 overexpression. (j) Western blotting was used to quantify COPS5 protein level in U2O3 and WEH1-231 cells after USP14 or UCH37 knockdown. (k) Western blotting was used to quantify COPS5 protein level in U2O3 and WEH1-231 cells after USP14 and UCH37 knockdown or treatment with b-AP15. (i, m) Expression and association of p53, USP14, UCH37 and COSP5 in primary tumor tissues from *p53*^+/−^ mice treated with DMSO (i, Ctrl, n=26) and b-AP15 (m, n=28).


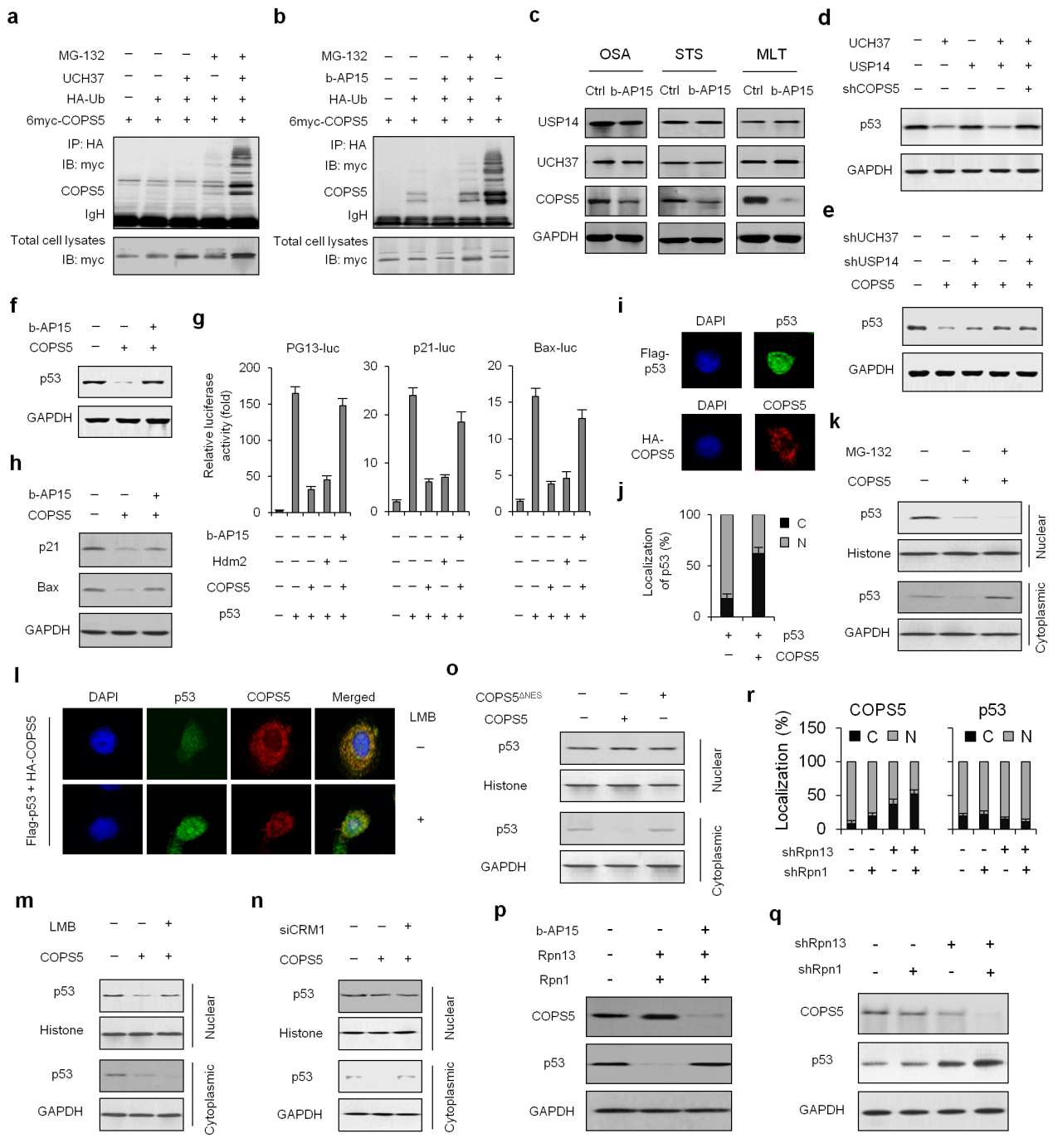


**Supplementary Figure 2. The effect of b-AP15 on p53-dependent regulation mechanism by UCH37 and USP14.** (a, b) COPS5 ubiquitination level in 293T cells after UCH37 overexpression (a) or treatment with b-AP15 (b). (c) USP14, UCH37 and COPS5 protein level in OSA, STS and MLT tissues from *p53*^+/−^ mice. (d-f) p53 prtein level was detected *in vitro* in 293T cells after UCH37 and USP14 overexpression (d), knockdown (e) or treatment with b-AP15 (f). (g) Luciferase activity assay of pG13, p21 or BAX reporter overexpressing Hdm2, COPS5, p53 and/or treated with b-AP15 in HeLa cells. (h) The protein level of p21 and BAX overexpressing COPS5 and/or treated with b-AP15 in HeLa cells. (i) Immunofluorescence analysis by either anti-HA and anti-Flag antibody after HA-COPS5 together with Flag-UCH37 expression plasmid was co-transfected in U2O3 cells. The nucleus was stained with DAPI (blue). (j) Fluorescence intensity of p53 in cytoplasm (C) and nucleus (N) was calculated using ImageJ 1.45. (k) The protein levels of p53 in cytoplasm and nucleus of U2O3 cells with treatment of MG-132 after transfection with vector control or COPS5 overexpression plasmids. (l) Immunofluorescence analysis by either anti-HA and anti-Flag antibody after HA-COPS5 together with Flag-UCH37 expression plasmid was co-transfected in U2O3 cells treated with DMSO or Leptomycin B (LMB). The nucleus was stained with DAPI (blue). (m-o) The protein levels of p53 in cytoplasm and nucleus of U2O3 cells with treatment of LMB (m), siCRM1 (n) or COPS5 (o) without nuclear export sequence (COPS5^ΔNES^) after transfection with vector control or COPS5 overexpression plasmids. Histone was used as a loading control for nuclear protein and GAPDH was used as a loading control for cytoplasmic protein. (p) The protein levels of p53 and COPS5 in U2O3 cells after transfection with vector control, Rpn13 and Rpn1 plasmids. (q) The protein levels of p53 and COPS5 in U2O3 cells with or without shRpn13 and shRpn1. (r) Gray intensity of Western blotting image for p53 and COPS5 in cytoplasm (C) and nucleus (N) of U2O3 cells with or without shRpn13 and shRpn1 was calculated using ImageJ 1.45.

**Table S1. The candidate UCH37-interacting proteins via Y2H screening.**

| **Symbol** | **Gene ID** | **Identity** | **Number** |
| --- | --- | --- | --- |
| Rpn13 | NM_007002.2 | 100% | 15 |
| Rpn10 | NM_002810.2 | 100% | 12 |
| COPS5 | NM_006837.2 | 100% | 10 |
| HAUS7 | NM_017518.6 | 98% | 11 |
| MLYCD | NM_012213.2 | 95% | 4 |
| PEX19 | NM_002857.3 | 92% | 8 |
| RANBP9 | NM_005493.2 | 89% | 6 |
| USP48 | NM_032236.5 | 85% | 4 |
| FAM58A | NM_152274.3 | 83% | 1 |
| RPS16 | NM_001020.4 | 76% | 1 |

**Table S2. The candidate UCH37-interacting proteins via co-IP and LC-MS/MS**

| **Symbol** | **UniProtKB** | **organism** |
| --- | --- | --- |
| Rpn13 | Q16186 | Homo sap |
| Rpn10 | P55036 | Homo sap |
| COPS5 | Q92905 | Homo sap |
| GRP78 | P11021 | Homo sap |
| UBOX4 | Q9UMS4 | Homo sap |
| KPYM | P14618 | Homo sap |
| DESP | P15924 | Homo sap |
| FILA | P20930 | Homo sap |
| GSTM3 | P21266 | Homo sap |
| SPB3 | P29508 | Homo sap |
| ARGI1 | P05089 | Homo sap |
| ANXA8 | P13928 | Homo sap |
| APRV1 | Q53RT3 | Homo sap |
| K2C80 | Q6KB66 | Homo sap |
| DUS14 | O95147 | Homo sap |
| S10A7 | P31151 | Homo sap |
| PKP1 | Q13835 | Homo sap |
| DSC3 | Q14574 | Homo sap |
